# High Expression of ERK-related RASGRF2 predicts Poor prognosis in patients with Stomach Adenocarcinoma and correlates with M2 macrophage

**DOI:** 10.7150/jca.63029

**Published:** 2021-10-22

**Authors:** Yaqi Du, Zhengguang Wang, Weina Wan

**Affiliations:** 1 Department of Gastroenterology, The First Affiliated Hospital of China Medical University, Shenyang, Liaoning, China.; 2 Department of Orthopedics, The First Affiliated Hospital of China Medical University, Shenyang, Liaoning, China.; 3 Department of Ultrasound, The First Affiliated Hospital of China Medical University, Shenyang, Liaoning, China.

**Keywords:** Immune infiltration, RASGRF2, Stomach adenocarcinoma, Nomogram, Immunoregulatory pathways

## Abstract

**Background:** The role of RASGRF2 has been verified in the development of various cancers. However, its roles in stomach adenocarcinoma (STAD) are still under investigation.

**Methods:** RASGRF2 transcript-level data and the associated clinical information from patients with STAD were extracted from The Cancer Genome Atlas (TCGA). Diagnostic and prognostic values of RASGRF2 were analyzed using receiver-operator characteristics (ROC) analysis, correlation analysis, and survival analysis in conjunction with a prognostic model. In addition, gene expression profiles, differentially-expressed genes for co-varying expression, and a differential expressed genes (DEG) protein-protein interaction network for influential nodes were also analyzed. To identify the molecular role of RASGRF2 in STAD, gene ontology (GO) term, Kyoto Encyclopedia of Genes and Genomes (KEGG) biological pathway, and gene set enrichment analysis (GSEA)-mediated functional module enrichment analyses were conducted. The relationship between RASGRF2 and gene signature-based predicted immune cell infiltration patterns were also investigated. To validate the bioinformatic findings, RASGRF2 protein expression was investigated *in vitro* using western blot and immunohistochemistry. Furthermore, relationships among RASGRF2 protein expression, clinicopathologic characteristics, and patient survival were analyzed.

**Results:** Bioinformatic analysis revealed a significantly higher RASGRF2 transcript level in STAD tissue, which was positively associated with the T stage, histological type, histological grade, and TP53 status. Moreover, the RASGRF2 transcript level indicated poor overall survival in STAD patients (hazard ratio = 1.47, *P* = 0.023). Multivariate Cox regression analysis showed that primary therapy outcome, age, and RASGRF2 transcript level were independent prognostic factors for survival, and the C-index of a nomogram was 0.695. Additionally, 159 genes were differentially expressed according to RASGRF2 transcript levels; 15 exhibited co-varying expression, and 13 were identified as influential nodes. The DEG-list was significantly enriched for several GO terms, biological pathways, and functional modules, including MAPK, RAS, ERK, and immunoregulatory pathways. RASGRF2 transcript levels were significantly positively correlated with infiltration levels of Tem, Macrophages, pDCs, and NK cells. Validation analysis showed similar results for the RASGRF2 protein expression level in both *in vitro* analyses.

**Conclusion:** Bioinformatic predictions combined with *in vitro* validation suggest that RASGRF2 plays diagnostic and prognostic roles and serves as a negative protective molecular factor in STAD patients.

## Introduction

Stomach adenocarcinoma (STAD) - one of the most common histological subtypes of stomach cancer - is highly aggressive, and its incidence and mortality rates have increased in recent years 1, 2. Despite a high five-year survival rate of up to 97 % if STAD is diagnosed at an early stage, the five-year survival rate drops to < 30 % in patients with advanced-stage STAD 3. Its etiology is complicated, and risk is influenced by both genetic and environmental factors (e.g. chronic *H. pylori* infection, salt consumption, nitrate and marinated food intake, obesity, and smoking) 4-6. Genetic factors are emerging as particularly significant due to the maturation of whole-genome sequencing technologies.

Protein RASGRF2 acts as an upstream regulator of the Ras-ERK signaling cascade and has been implicated in malignant mesothelioma (MM) risk by a genome-wide association study 7. High RASGRF2 expression regulates MMP9 levels via modulation of the Src/PI3-kinase and NF-κB pathways and inhibits migration and invasion in colorectal cancer (CRC) 8. A previous study suggested that reciprocal ANXA6 and RASGRF2 expression could delineate rapidly growing from invasive triple-negative breast cancer 9. Interestingly, Calvo *et al.* (2011) found that RASGRF2 could prevent Cdc42 activation, thus inhibiting Cdc42-mediated cellular processes, including transformation, cytoskeletal dynamics, and tumor cell motility. The role of RASGRF2 in the negative regulation of Cdc42 may partly explain its protective function in the context of cancer 10. Moreover, RASGRF2 expression is reportedly regulated by β-arrestin-1, thereby modulating membrane protrusion, cell migration, and invasion 11. While RASGRF2 demonstrates potent antineoplastic activity across a variety of tissues and cancers, few studies have focused on its role in STAD. Therefore, the present study aimed to investigate the potential role of RASGRF2 in STAD, including functional mechanisms and diagnostic as well as prognostic utility.

## Materials and methods

### Date acquisition and Pan-cancer analysis of *RASGRF2*

To determine the expression level of RASGFR2 in normal healthy tissues, adjacent tumor samples, and tumor samples, TPM-normalized expression data 12 for RASGRF2 from The Cancer Genome Atlas (TCGA) Pan-cancer 13 and GTEx datasets were downloaded from the UCSC XENA dataset (https://xenabrowser.net/datapages/), which included 31 types of tumors and relevant normal tissues. We performed the Wilcoxon rank-sum test to compare RASGRF2 expression among different cancer and paired normal tissue samples.

### RASGRF2 differential expression analysis and assessment of RASGRF2 transcript level diagnostic performance

STAD TPM-normalized expression data profiles and relevant clinical data were downloaded from the UCSC cancer browser. We performed a comparison of RASGRF2 transcript levels between STAD (TCGA) and adjacent normal (TCGA + GTEx) tissues. In addition, receiver-operator characteristics (ROC) curves were constructed to evaluate the efficacy of the *RASGRF2* transcript level by using the pROC package 14. An area under the curve (AUC) value ranging from 0.5 to 1.0 indicates the discrimination ability from 50 to 100%.

### Correlation between *RASGRF2* transcript level and clinicopathologic characteristics of patients with STAD

The correlation between TCGA *RASGRF2* transcript level (classified as 'high' or 'low' based on median transcript level) and clinicopathologic characteristics of samples from patients with STAD was analyzed using the χ^2^ test, Fisher's exact test, the Kruskal-Wallis rank-sum test, and the Wilcoxon rank-sum test.

### Survival analysis, prognostic model generation, and construction and validation of a nomogram

The difference in overall survival (OS) between high and low *RASGRF2* transcript level groups was determined using Kaplan-Meier survival analysis as provided in the *survminer* package 12. Univariate was followed by multivariate Cox regression analysis to construct the optimal prognostic model: variables (including *RASGRF2* transcript level and clinicopathologic characteristics) achieving *P* < 0.05 during univariate analysis were included in the multivariate Cox regression model. The model was then used to predicting survival, and the predicted value was compared to the actual observed value.

Independent prognostic factors (including the *RASGRF2* transcript level) identified by multivariate Cox regression analysis informed the construction of a nomogram for the prediction of survival probability. The TCGA STAD cohorts were randomly divided into a “proper” training set and a “calibration” set. The rms package (https://cran.r-project.org/web/packages/rms/index.html) was used to construct the survival prediction model. Calibration curves were constructed to graphically evaluate the agreement of predicted and observed survival rates. The consistency (C)-index (typically ranging from 0.5 to 1) was calculated to determine nomogram predictive power.

### Global differential expression analysis, co-variation of significantly differentially expressed genes (DEGs), protein-protein interaction (PPI) network construction, and identification of influential nodes

The *DESeq2* package 15 was used to perform differential expression analysis of HTSeq-count data between high and low *RASGRF2* transcript level groups. Differences were considered significant at |log_2_FC| > 2.0 and adjusted *P* < 0.05. The STRING database (https://string-db.org) (version 11.0) 16, in conjunction with Cytoscape (version 3.7.1) 17, was used to construct the DEG PPI network. Any interaction with a combined score > 0.4 was considered significant. The Molecular Complex Detection (MCODE) plug-in (version 1.5.1) 18 was used to identify significant gene modules based on the following selection criteria: MCODE scores > 5, degree cut-off = 2, node score cut-off = 0.2, maximum depth = 100, and k-score = 2. Nodes were considered as hub genes with degree ≥8.

### Functional enrichment analyses and gene set enrichment analysis (GSEA)

To determine which RASGRF2-relevant biological functions and pathways may underlie its associations with STAD. The *ClusterProfiler* package (http://bioconductor.org/packages/release/bioc/html/clusterProfiler.html) (version 3.8.0) 19 was used to perform gene ontology (GO) term and Kyoto Encyclopedia of Genes and Genomes (KEGG) biological pathway enrichment analysis using the list of genes differentially expressed between high and low *RASGRF2* transcript level groups 20, 21. Furthermore, based on the expression gene sets of RASGRF2, GSEA (http://software.broadinstitute.org/gsea/index.jsp) functional module enrichment analysis 22, 23 was performed. Finally, the *ClusterProfiler* package was also used to determine which functional modules were significantly enriched. The gene set collections from the Molecular Signatures Database (MSigDB, version 3.0) 24 were used for differential gene set enrichment analysis. Enrichment was considered significant at a false discovery rate (FDR) of < 0.25, an adjusted *P* < 0.05, and Normalized Enrichment Score (NES) > 1.

### Immune infiltration profiling using single-sample GSEA (ssGSEA)

The ssGSEA method provided by the *GSVA* package (http://www.bioconductor.org/packages/release/bioc/html/GSVA.html) 25 was used to estimate relative tumor infiltration levels of 24 immune types; observed gene expression levels were parsed for known immune gene signatures 7. Spearman correlation was used to explore associations between *RASGRF2* transcript level and ssGSEA-estimated immune infiltration level. Correlations were considered significant at *P* < 0.05 and |R|≥ 0.4. The Wilcoxon rank-sum test was used to analyze differences in immune infiltration levels between high and low *RASGRF2* transcript level groups. Differences were considered significant at *P* < 0.05.

### Validation cohort assembly and specimen collection

Primary tumor samples were collected from 72 patients with gastric cancer undergoing surgery at The First Affiliated Hospital of China Medical University between January and September 2010. Study protocols were approved by the Ethics Committee of The First Affiliated Hospital of China Medical University (AF-SOP-07-1.1-01). All participants provided written informed consent. Patients diagnosed with gastric cancer without other serious diseases were enrolled in the study. During surgery, 72 samples of tumor tissue, peritumoral tissue (within 3 cm of the tumor edge), and gastric normal tissue (3 cm from the tumor edge) were collected from the 72 patients and stored at -80°C for future use. The inclusion criteria were used as follows: (1) patients pathologically confirmed with gastric cancer; (2) patients subjected to surgery; (3) patients aged 18-80 years. The exclusion criteria included receiving neoadjuvant chemotherapy or radiotherapy, remnant gastric cancer, and postoperative death within 3 months. The pathological diagnoses and classifications were estimated according to the AJCC Cancer Staging Manual (7th edition)^26^. In the collected 72 STAD patients, there were 52 males and 20 females. The histopathologic subtypes of the 72 STAD were classified into Papillary type (n = 3, 4.2%), tubular type (n = 17, 23.6%), poorly differentiated type (n = 27, 37.5%), signet ring type (n = 16, 22.2%), and mucinous type (n = 9, 12.5%). Detailed clinicopathological features of STAD patients were shown in **Table 1**.

### Immunohistochemistry (IHC)

To determine the differences between RASGRF2 protein expression levels in tumor and adjacent non-tumor tissue and whether the RASGRF2 protein expression correlated with other clinicopathologic characteristics, IHC was performed on validation cohort tumor and adjacent non-tumor tissue specimens. All tissue specimens were fixed in neutral formaldehyde, embedded in paraffin, and sectioned (thickness, 4 μm). The streptavidin-peroxidase immunohistochemical method was used to enhance staining intensity. Tissue sections were incubated at 4 °C overnight with anti-RASGRF2 (1:100) (ab121577; rabbit anti-human; mono-clonal; Abcam Inc, Cambridge, United Kingdom), and phosphate-buffered saline was used as a blank control. Sections were then incubated with a goat anti-rabbit secondary antibody (1:200) (G1213; monoclonal; Servicebio Inc, Wuhan, China) at 37 °C for 30 minutes, followed by diaminobenzidine for color development. Finally, samples were lightly counterstained with hematoxylin, dehydrated in alcohol, and mounted. Two investigators blinded to the clinical data semiquantitatively scored the slides by evaluating the staining intensity and percentage of stained cells in representative areas. The staining intensity was scored as 0 (negative), 1 (weak), 2 (moderate), or 3 (strong). The percentage of cells stained was scored as 1 (1-25%), 2 (26-50%), 3 (51-75%), or 4 (76-100%). A final combined score between 0 and 12 was obtained by multiplying intensity and percentage scores. Specimens with scores of > 3 were considered RASGRF2-positive and those with scores > 5 indicating strong positive expression. Patients were classified into high or low RASGRF2 protein expression groups based on median scores. The t-test (Two-tailed) was used to compare RASGRF2 protein expression between tumor and non-tumor tissue specimens. The Pearson χ^2^ test was used to test differences in clinicopathologic characteristics between high and low RASGRF2 protein expression groups.

### Validation cohort Kaplan-Meier survival analysis

In order to assess the RASGRF2 protein expression prognostic value, the difference in OS between high and low RASGRF2 protein expression groups was determined using Kaplan-Meier survival analysis (as provided in the *survival* package) 27 in conjunction with the Wilcoxon log-rank test.

### Cell culture

To further validate that RASGRF2 transcript-level observations translate to the protein level, western blotting was performed using representative cell lines. One human gastric epithelial cell line (GES-1) and three human gastric cancer (GC) cell lines (AGS, HGC-27, and KATO III) were purchased from China National Infrastructure of Cell Line Resource (Beijing, China). Culture media were as follows: Dulbecco's Modified Eagle's Medium (DMEM; HyClone Inc., Logan, Utah, USA) for GES-1, F12 medium (HyClone Inc., Logan, Utah, USA) for AGS, Roswell Park Memorial Institute (RPMI)-1640 medium (HyClone Inc., Logan, Utah, USA) for HGC-27, and Iscove's Modified Dulbecco's Medium (IMDM; Thermo Fisher Scientific Inc., Logan, Utah, USA) for KATO III. All media were supplemented with 10% fetal bovine serum (FBS; HyClone Inc., Logan, Utah, USA).

### Western blotting

Total protein was extracted using a RIPA lysis buffer (P0013C; Beyotime Inc., Shanghai, China) and quantified using the Bradford method. In total, 30 μg of protein lysates were separated using sodium dodecyl sulfate-polyacrylamide gel electrophoresis (8% resolving gel) and electroblotted onto polyvinylidene fluoride membranes (Merck Millipore, Billerica, MA, USA). Membranes were incubated overnight at 4 °C with the following primary antibodies: anti-RASGRF2 (1:500) (ab121577; rabbit anti-human; mono-clonal; Abcam Inc., Cambridge, England) and anti-β-tubulin (1:1000) (#2146; rabbit anti-human; mono-clonal; Cell Signaling Inc, Danvers, MA, USA). Membranes were washed and subsequently incubated with Goat Anti-Rabbit IgG (H + L)-HRP Conjugate (1:8000) (#1706515; Bio-Rad Laboratories Inc, Hercules, CA, USA) at 37 °C for 2 hours. Bound proteins were visualized using an ECL Gel Imaging System (MF-Chemibis 2.0, Thermo, USA). Between-group RASGRF2 protein expression levels were compared using the t-test (Two-tailed).

### Statistical analysis

R (v.3.6.2) was used only for all statistical analyses 15. The Wilcoxon rank-sum test was used to compare RASGRF2 expression among different cancer and paired normal tissue samples and between STAD (TCGA) and adjacent normal (TCGA + GTEx) tissues. The χ^2^ test, Fisher's exact test, the Kruskal-Wallis rank-sum test, and the Wilcoxon rank-sum test were used to evaluate correlations between TCGA *RASGRF2* transcript level and the clinicopathologic characteristics of samples from patients with STAD. 'Exact' means that the statistical method used was the Fisher's exact test. Kaplan-Meier survival analysis and both univariate and multivariate Cox regression analyses were used to evaluate the prognostic utility and construct a prognostic model. Multivariate Cox analysis incorporated estimation of individual factor hazard risk (HR), including 95% confidence intervals (CIs). *P* < 0.05 was considered statistically significant in all tests.

## Results

### Characteristics of patients from the TCGA STAD dataset

Patient clinical characteristics and *RASGRF2* transcript level data for 375 primary tumors are shown in **Table S1**. Samples without the corresponding clinical information were not included in the next statistical analysis. The patients were divided into two groups with relatively low (188 cases) and high (187 cases) RASGRF2 expression groups in STAD. There were 134 females (35.7%) and 241 males (64.3%) in the cohort. The percentage of patients younger than 65 years old was 43.7% and that of patients up to 65 years old was 55.2%. According to the TNM stage, 246 (68.9%) cases out of 357 had regional lymph node invasion and 25 (7%) out of 355 had distant metastases. Concerning the histological type, 63 (16.9%) were diffuse type, 19 (5%) were mucinous type, 207 (55.3%) were not otherwise specified, 5 (1.3%) were papillary type, 11 (2.9%) were signet ring type, and 63 (18.4%) were the tubular type. With respect to histological grade, 10 (2.7%) were G1, 137 (37.4%) were G2, and 219 (59.8%) were G3. Regarding TP53 status, 172 (46.2%) cases were Mut and 200 (53.8%) were WT. Finally, with regard to the PIK3CA status, 59 (15.9%) cases were Mut and 313 (84.1%) were WT. Mut, mutant; WT, wild-type.

### Differential expression of *RASGRF2* transcript level in tumor versus normal tissue, correlation with clinicopathologic characteristics, and assessment of RASGRF2 transcript level diagnostic utility in TCGA

**Figure 1:**
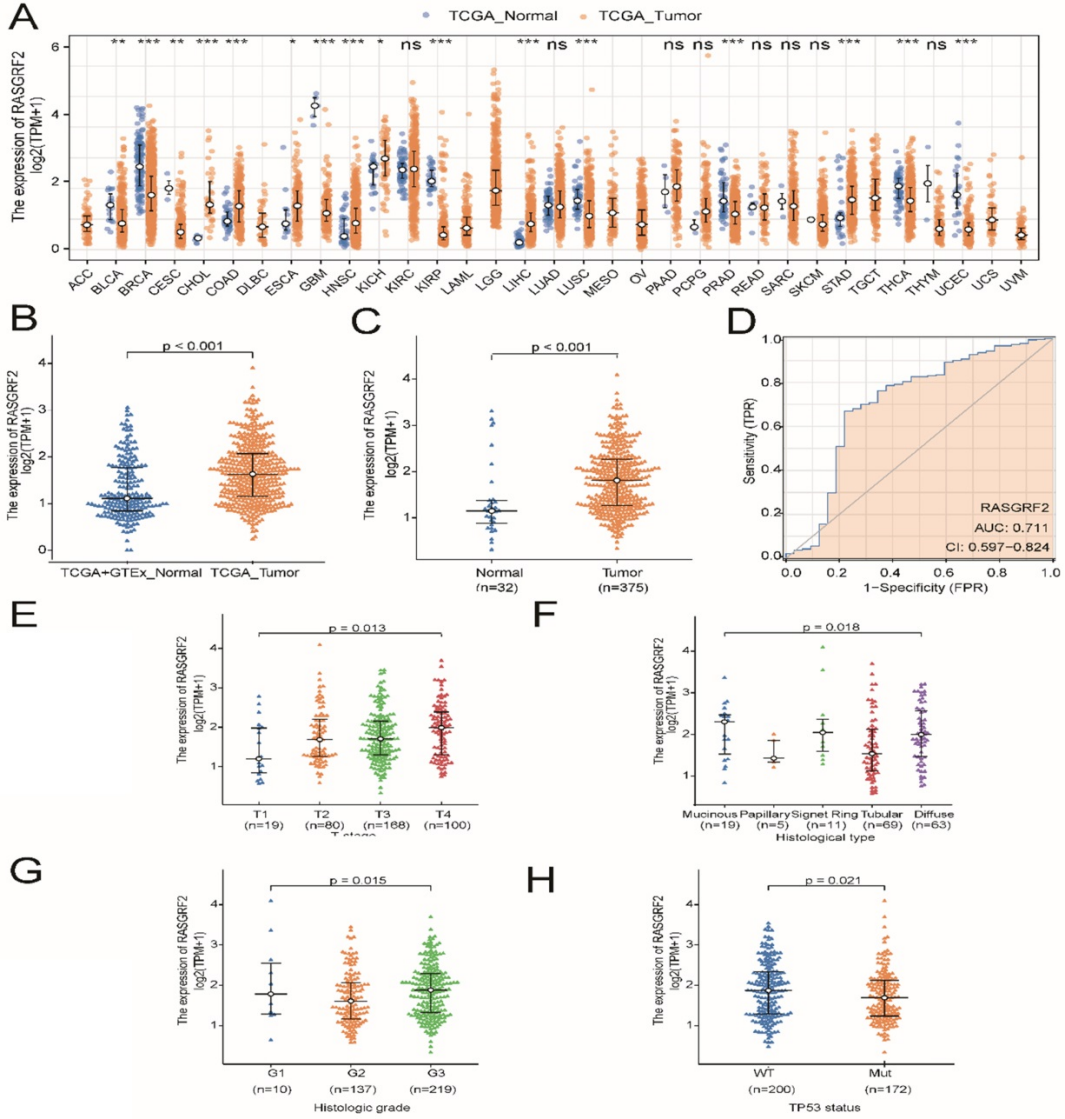
** Diagnostic utility of *RASGRF2* transcript level and correlation with clinicopathologic characteristics (based on The Cancer Genome Atlas (TCGA) stomach adenocarcinoma (STAD) dataset). (A)** Comparison of *RASGRF2* transcript levels between all tumor subtype (TCGA) and normal (TCGA + GTEx) tissues. **(B)** Comparison of *RASGRF2* transcript levels between STAD tumor (TCGA) and normal (TCGA + GTEx) tissues. **(C)** Comparison of *RASGRF2* transcript levels between TCGA STAD tumor and adjacent non-tumor tissues. **(D)** Receiver-operator characteristics curve. **(E-H)** Correlation of *RASGRF2* transcript level with STAD clinicopathologic characteristics including T stage (E), histologic type (F), histologic grade (G), and *TP53* status (H). (*P ≤0.05, **P≤0.01, ***P≤0.001).

Expression of *RASGRF2* was significantly up-regulated in most TCGA dataset tumor subtypes (relative to TCGA + GTEx dataset normal tissue), including STAD (*P* < 0.001) (**Fig. 1A-B**), and was also significantly up-regulated in TCGA dataset STAD tumor tissue (relative to adjacent non-tumor tissue) (*P* < 0.001) (**Fig. 1C**). Regarding the ability of *RASGRF2* expression to discriminate between patients with STAD and healthy individuals, the ROC area under the curve was 0.711 (**Fig. 1D**). Moreover, high *RASGRF2* expression correlated significantly with T stage (*P* = 0.013), histologic type (*P* = 0.018), histologic grade (*P* = 0.015), and *TP53* status (*P* = 0.021) (based on Kruskal-Wallis and Wilcoxon rank-sum tests) (**Fig. 1E-H**). An independent correlation analysis based on χ^2^ and Fisher's exact tests agreed that high *RASGRF2* expression correlated significantly with T stage (*P* = 0.033), histological type (*P* = 0.020), and *TP53* status (*P* = 0.028), but not with histologic grade (*P* = 0.053) (**Table S1**).

### Survival analysis, prognostic model generation, and nomogram construction and validation

**Figure 2:**
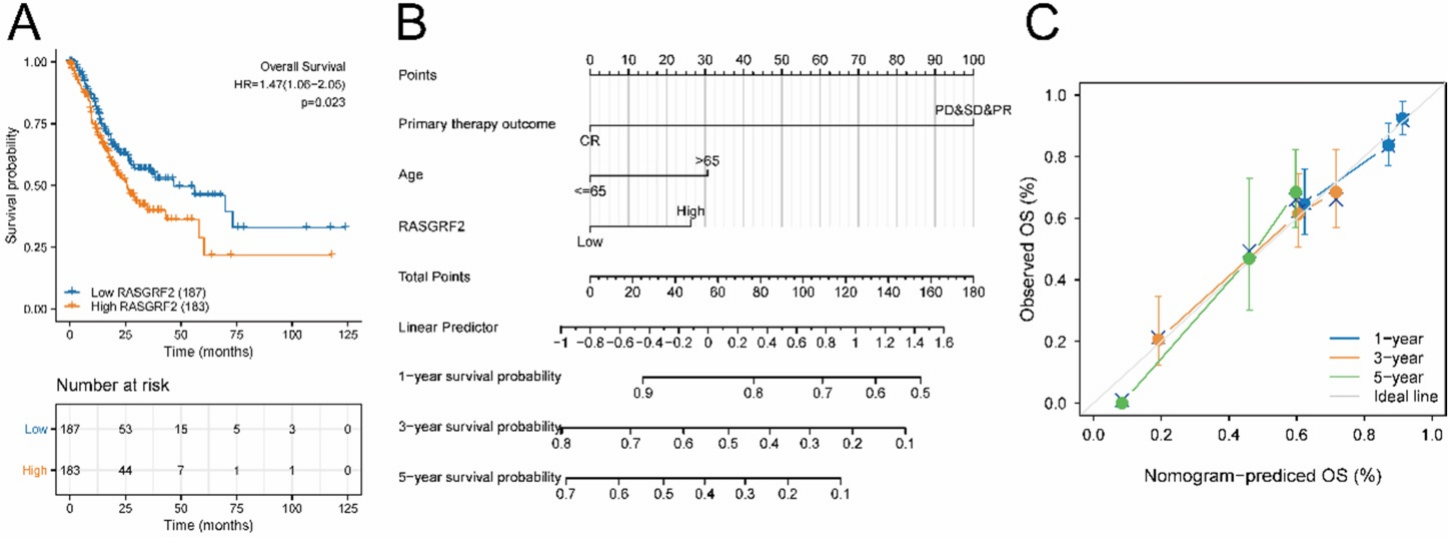
** Prognostic significance of *RASGRF2* transcript level. (A)** Kaplan-Meier survival analysis for patients with stomach adenocarcinoma exhibiting high versus low *RASGRF2* transcript levels (*P* = 0.023). **(B)** A five-year nomogram based on prognostic risk factors was identified by multivariate Cox regression. **(C)** Nomogram calibration plots.

The high *RASGRF2* expression group exhibited significantly poorer OS (relative to the low *RASGRF2* expression group) (HR = 1.47; 95% CI [1.06, 2.05]; *P* = 0.023) (**Fig. 2A**). Multivariate Cox regression indicated that primary therapy outcome (HR = 0.233; 95% CI [0.149, 0.365]; *P* < 0.001), age (HR = 1.699; 95% CI [1.100, 2.624]; *P* = 0.017), and *RASGRF2* expression level (HR = 1.550; 95 % CI [1.006, 2.390]; *P* = 0.047) were prognostic factors independently correlated with poor OS (**Table S2**). Using the nomogram constructed based on these three risk factors, primary therapy outcome was found to contribute the greatest number of risk points (ranging from 0 to 100). The nomogram C-index was 0.695 (95% CI [0.671, 0.718]) (**Fig. 2B**), and the calibration plot bias-corrected line was found to be close to the ideal (i.e. the 45-degree line) (**Fig. 2C**).

### Identification of DEGs between high and low *RASGRF2* expression groups, analysis to identify DEGs with co-varying expression, and identification of influential nodes in the DEG PPI network

**Figure 3:**
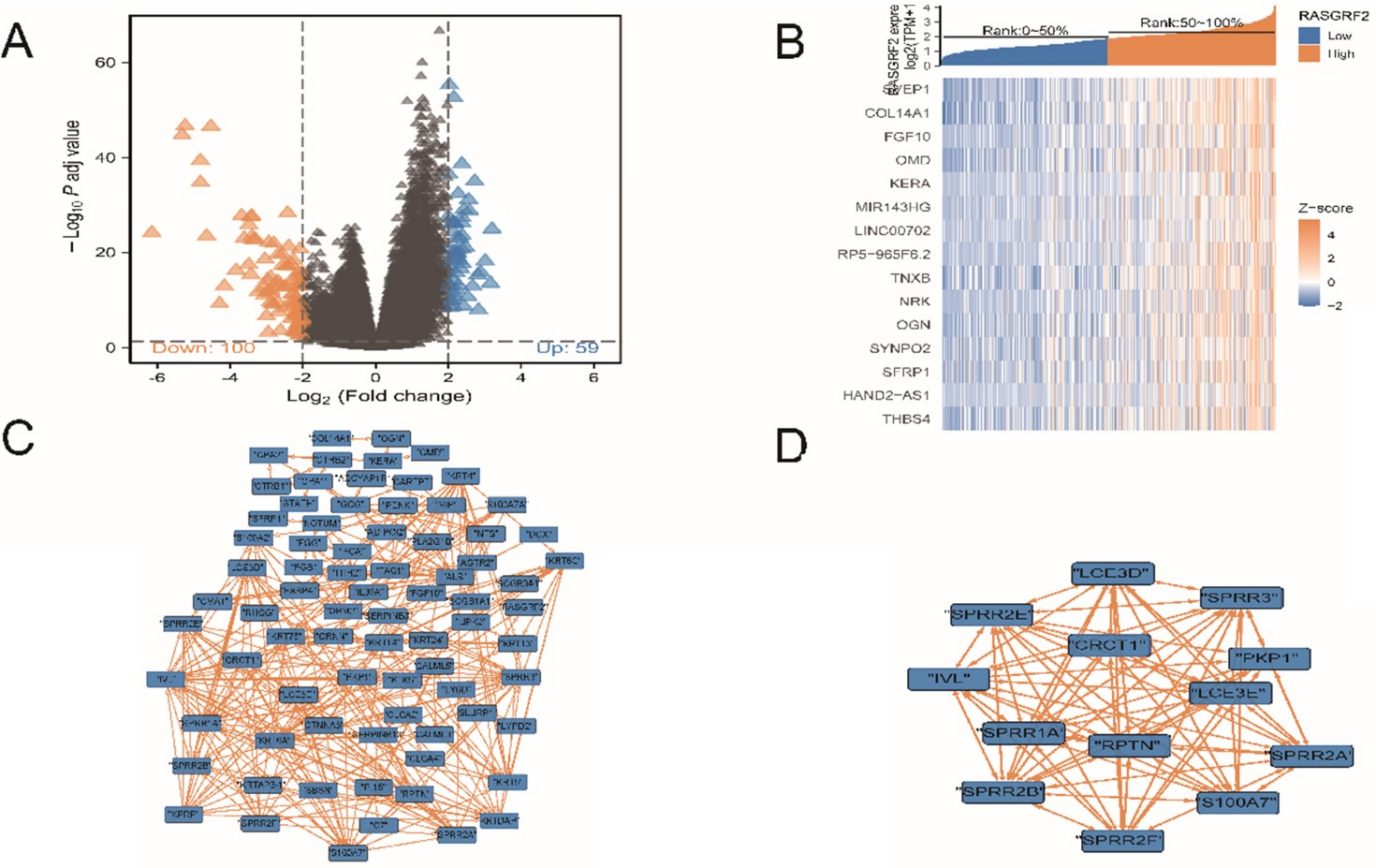
** Differentially-expressed genes (DEGs), co-variation of DEG expression, DEG protein-protein interaction (PPI) network, and influential network nodes. (A)** Volcano plot demonstrating 159 DEGs. **(B)** Heat map demonstrating expression levels of 15 DEGs with co-varying expression. **(C)** DEG PPI network. **(D)** Thirteen influential network nodes (hub genes).

A total of 159 DEGs were identified, of which 59 were up-regulated and 100 were down-regulated (**Fig. 3A**). Fifteen DEGs exhibited co-varying expression (**Fig. 3B**): *SVEP1*, *COL14A1*, *FGF10*, *OMD*, *KERA*, *MIR143HG*, *LINC00702*, *RP5-965F6.2*, *TNXB*, *NRK*, *OGN*, *SYNPO2*, *SFRP1*, *HAND2-AS1*, and *THBS4*. Within the DEG PPI network (**Fig. 3C**), a total of 13 nodes were identified as hubs (**Fig. 3D**); gene names and functions are provided in **Table S3**.

### Enrichment analyses and GSEA analysis-based identification of RASGRF2-related functional modules

**Figure 4:**
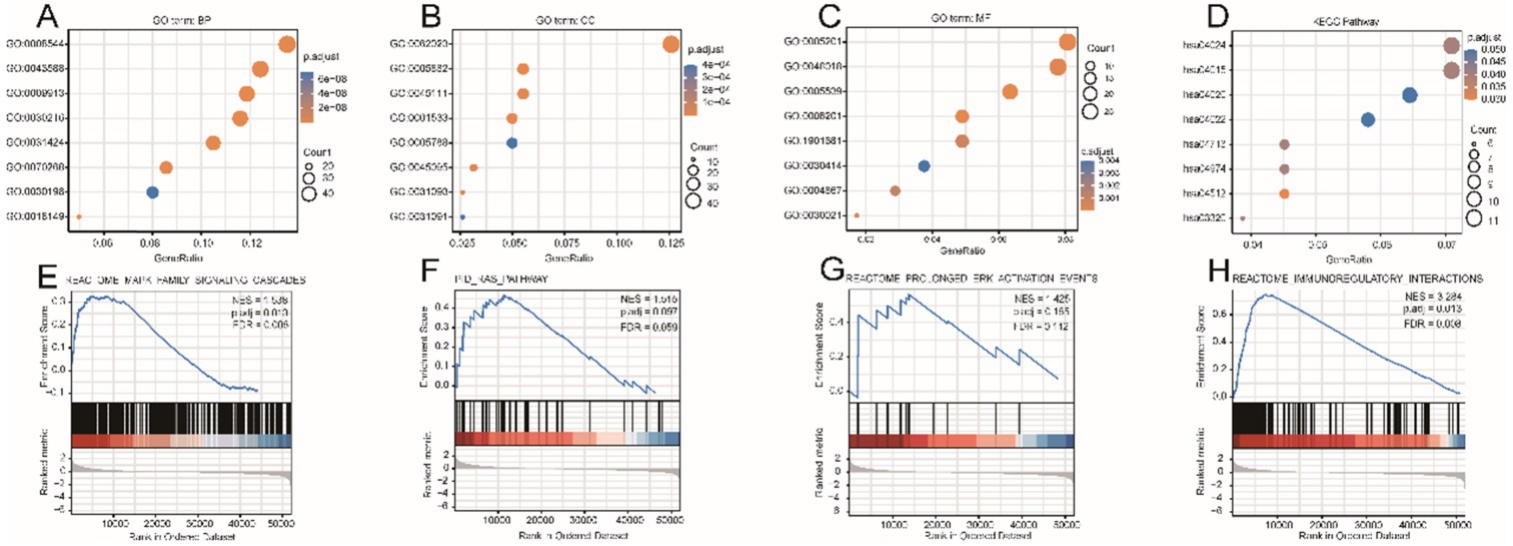
** Gene ontology (GO), Kyoto Encyclopedia of Genes and Genomes (KEGG) biological pathway, and gene set enrichment analysis (GSEA)-based functional module enrichment analyses of differentially expressed genes. (A-C)** Enriched GO biological process, cellular component, and molecular function terms. **(D)** Enriched KEGG pathways. **(E-H)** Enrichment plots indicating enriched GSEA functional modules, including MAPK family, RAS, ERK, and immunoregulatory signaling pathways. Abbreviations: ES enrichment score, NES normalized ES, NOM *P*-Val normalized *P*-value.

The DEG-list was significantly enriched for various biological processes (BPs) such as epidermis development, epidermal cell differentiation, and skin development; molecular functions (MFs) such as extracellular matrix structural constituent, receptor-ligand activity, and glycosaminoglycan binding; and cell components (CCs) such as collagen-containing extracellular matrix, intermediate filament cytoskeleton, and intermediate filament (**Fig. 4A-C**). Signaling pathways such as Rap1 (hsa04015), cAMP (hsa04024), Calcium (hsa04020), and cGMP-PKG (hsa04022) were highly enriched in the KEGG pathways (**Fig. 4D**). Other details of these GO terms and KEGG pathways are shown in Table S4. The results of GSEA indicated that four functional modules were significantly enriched in the high *RASGRF2* expression group: MAPK family (NES = 1.538, adjusted *P* = 0.013, FDR = 0.008), RAS (NES = 1.515, adjusted *P* = 0.097, FDR = 0.059), ERK (NES = 1.425, adjusted *P* = 0.185, FDR = 0.112), and immunoregulatory (NES = 3.284, adjusted *P* = 0.013, FDR = 0.008) (**Fig. 4E-H**). This indicates that RASGRF2 expression may be associated with the altered functioning of such signaling pathways, thereby suggesting potential mechanisms by which RASGRF2 may play a role in STAD pathogenesis.

### Association between *RASGRF2* transcript level and immune infiltration pattern

**Figure 5:**
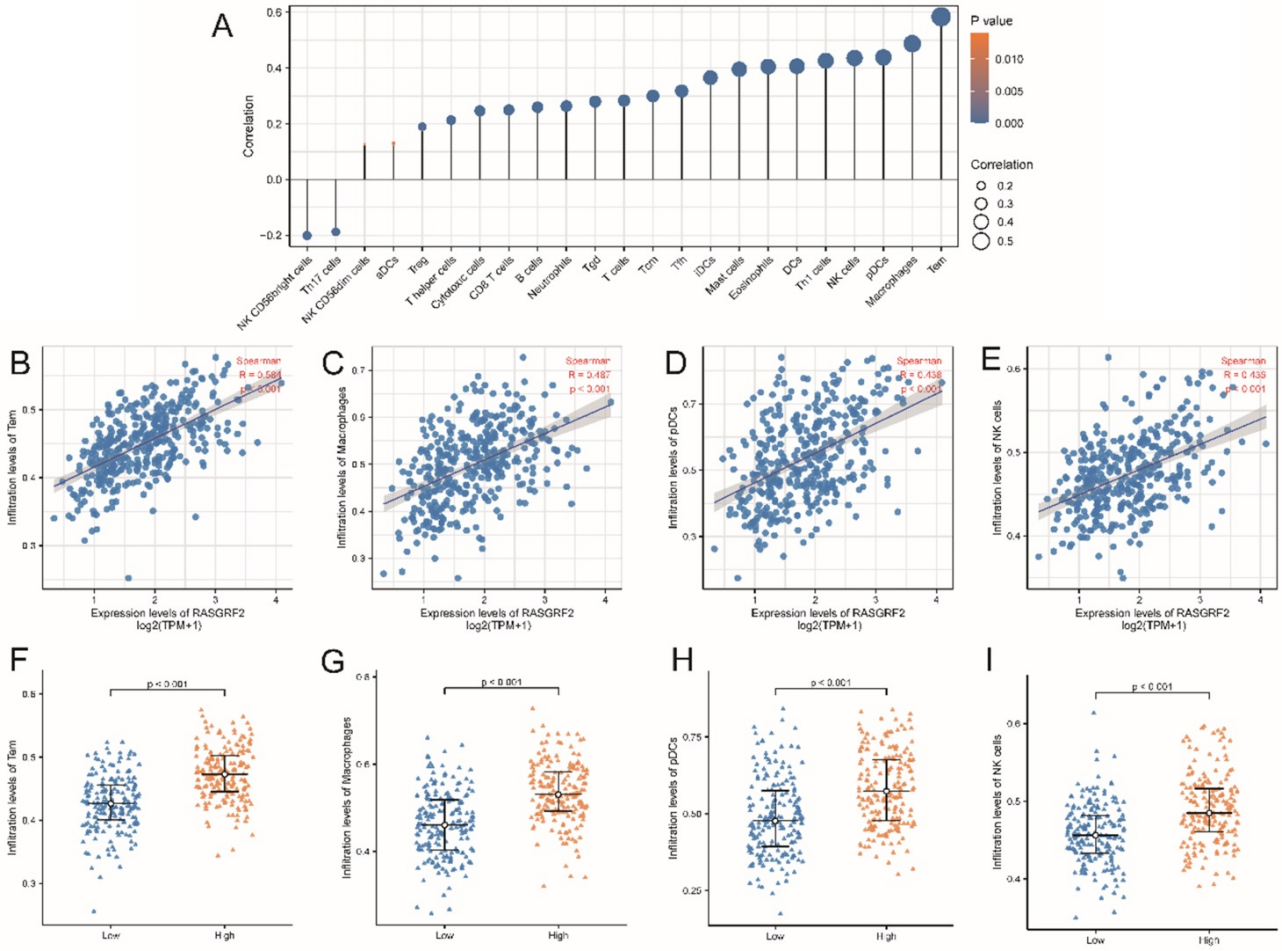
** Correlation between *RASGRF2* transcript level and Immune infiltration pattern. (A)** Varying predicted tumor infiltration proportions of 24 immune subtypes. **(B-E)** Correlations between *RASGRF2* transcript level and predicted immune infiltration levels. **(F-I)** Comparison of immune infiltration levels between high and low *RASGRF2* transcript level groups (*P* < 0.001).

Predicted infiltration by most immune cell types was correlated with *RASGRF2* expression (**Fig. 5A**). For example, infiltration by Tem (R = 0.584, *P* < 0.001), macrophages (R = 0.487, *P* < 0.001), pDCs (R = 0.438, *P* < 0.001), and NK cells (R = 0.436, *P* < 0.001) was significantly positively correlated with *RASGRF2* expression (**Fig. 5B-E**). Enrichment scores for Tem (*P* < 0.001), macrophages (*P* < 0.001), pDCs (*P* < 0.001), and NK cells (*P* < 0.001) were significantly higher in the high (relative to the low) *RASGRF2* expression group (**Fig. 5F-I**).

### Validation of RASGRF2 protein expression, correlation with clinicopathologic characteristics, and prognostic model performance in an independent STAD cohort

**Figure 6:**
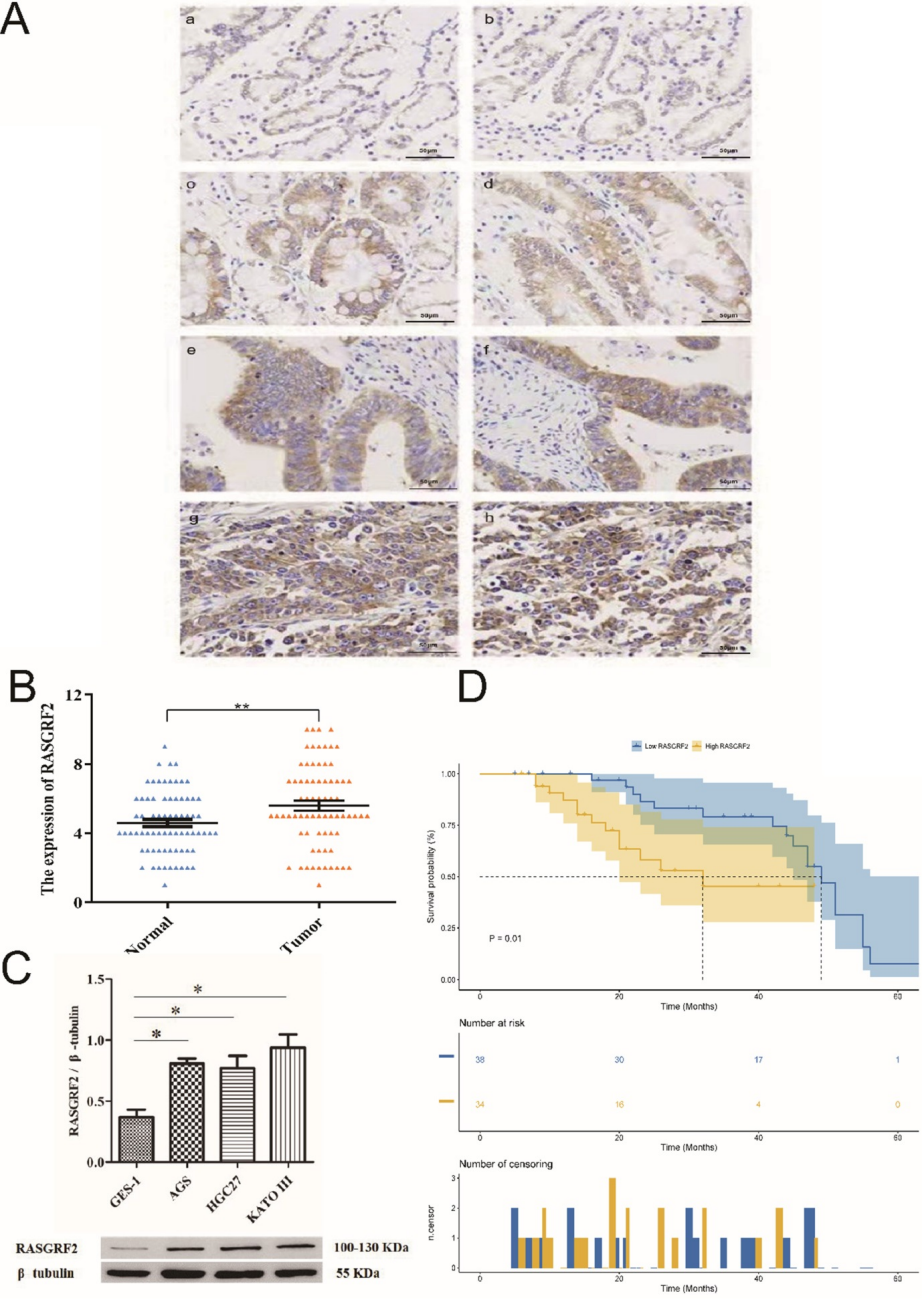
** RASGRF2 protein expression levels and prognostic significance in tissue specimens from a stomach adenocarcinoma-validation cohort. (A)** Immunohistochemistry demonstrating RASGRF2 protein expression level and subcellular localization in STAD and adjacent non-tumor tissue specimens. **(B)** Staining for RASGRF2 was significantly more intense in STAD tissue than that in adjacent non-tumor tissue (*P* = 0.0051). **(C)** Kaplan-Meier survival analysis demonstrating significantly different survival between patients with STAD exhibiting high versus those with low RASGRF2 protein expression (*P* = 0.01). **(D)** Western blotting demonstrating significantly differential RASGRF2 protein expression between the GES-1 cell line and three gastric cancer cell lines (AGS, HGC-27, and KATO III) (*P* < 0.05).

Mean age of the independent cohort was 59 years (minimum age: 36 years, maximum age: 79 years) and it included both male (n = 52, 72.2%) and female (n = 20, 27.8%) patients. Patients at clinical stages I (n = 4, 5.6%), II (n = 27, 37.5%), III (n = 38, 52.8%), and IV (n =3, 4.2%) were all present. Papillary type (n = 3, 4.2%), tubular type (n = 17, 23.6%), poorly differentiated type (n = 27, 37.5%), signet ring type (n = 16, 22.2%), and mucinous type (n = 9, 12.5%) were all represented. Both STAD tissue specimens (84.7% [61/72]) and adjacent non-tumor tissue specimens (86.1% [62/72]) exhibited cytoplasmic RASGRF2. However, in agreement with TCGA cohort findings, RASGRF2 staining was significantly more intense in STAD tissue specimens (score > 5 in 70.8 % (51/72)) (**Fig. 6Ac-h**) than in adjacent non-tumor tissue specimens (score > 5 in 47.2 % (34/72)) (**Fig. 6Aa-b**) (*P* = 0.0051) (**Fig. 6B**) (**Table 1**). Moreover, in agreement with TCGA cohort findings, high RASGRF2 protein expression correlated positively with clinical stage (*P* = 0.009), T stage (*P* = 0.042), and histological type (*P* = 0.032) (**Table 1**), and Kaplan-Meier survival analysis demonstrated significantly poorer OS in patients exhibiting high RASGRF2 protein expression (*P* = 0.01) (**Fig. 6D**). Finally, multivariate Cox regression identified age (HR = 1.359; 95% CI [1.141, 1.911]; *P* = 0.031), N stage (HR = 1.549; 95% CI [1.323, 1.933]; *P* = 0.026), and RASGRF2 protein expression level (HR = 1.239; 95% CI [1.069, 1.823]; *P* =0.023) as independent prognostic factors predicting OS (**Table 2**). All statistics shown in Tables 1 and 2 using the χ^2^ test and t-test (two-tailed) were analyzed using SPSS statistical software (version 23.0.0) in **Fig. 6B**.

### Validation of RASGRF2 protein expression in cell lines

Expression of RASGRF2 protein was significantly up-regulated in the three GC cell lines (AGS, *P* = 0.0029; HGC-27, *P* = 0.015; KATO III, *P* = 0.004) relative to the GES-1 cell line (**Fig. 6C**). The t-test (two-tailed) were analyzed using SPSS statistical software (version 23.0.0).

## Discussion

The RASGRF2 protein belongs to the family of Ras guanine nucleotide releasing factors and functions as a calcium-regulated exchange factor 28. Prior research has established the importance of RASGRF2 across a diverse range of physiological and pathological conditions. Up-regulated expression of RASGRF2 is closely associated with hippocampal neuron formation in the neonate 29, contributes to the diagnosis of triple-negative breast cancer 30, and is implicated in regulating cell polarity and tumorigenesis 31. The present study investigated the potential diagnostic and prognostic value of RASGRF2 expression level in the context of STAD, including potential underlying molecular mechanisms.

Findings indicated that *RASGRF2* transcript levels were higher in STAD relative to normal gastric tissue, as well as in STAD tumors relative to adjacent non-tumor tissue. This was validated at the protein level both *in vitro* and clinically by demonstrating that RASGRF2 protein was highly expressed in three STAD-relevant cell lines (relative to a normal gastric cell line) and in tumor relative to adjacent non-tumor tissue in an independent STAD cohort. Public dataset and independent cohort findings, respectively, demonstrated that high *RASGRF2* transcript levels and high RASGRF2 protein expression levels correlate significantly and positively with clinicopathologic measures (e.g. clinical-stage, T stage, and histological type) of STAD severity. Findings of a ROC analysis suggested that RASGRF2 may have diagnostic value as a biomarker in the context of STAD, while findings of a Kaplan-Meier survival analysis in conjunction with a multivariate Cox regression model suggested that RASGRF2 may also have prognostic value (e.g. in predicting OS). This is consistent with previous data demonstrating the ability of RASGRF2 to discriminate between rapidly-growing and invasive triple-negative breast cancer subtypes 9.

To investigate potential molecular mechanisms underlying the association between RASGRF2 expression and STAD, GO term and biological pathway enrichment analysis as well as GSEA were conducted, using as inputs the list of genes significantly differentially expressed in patients with STAD exhibiting high *RASGRF2* transcript levels (and gene expression levels, in the case of GSEA). Four significantly enriched functional modules were identified by GSEA, including three key signaling pathways: MAPK family, RAS, and ERK. The MAPK signaling pathway is reportedly essential for cell proliferation, and suppression of this pathway inhibited gastric cancer cell proliferation and growth 32. Similarly, MAPK pathway activation has been implicated in the resistance of gastric cancer to apatinib 33. After analyzing genomic DNA from 104 gastric tumors, Yoo *et al.* (2002) found that *RAS* mutations are uncommon among gastric adenocarcinomas, but that elevated ERK1/2 activity was a characteristic of invasive tumors 34. Recent studies have furthermore demonstrated that inactivation of the Ras/Raf/MEK/ERK pathway attenuates gastric carcinogenesis in nude mice 35. Indeed, the extracellular signal-regulated kinase (ERK) cascade (a.k.a. Ras/Raf/MEK/ERK or simply Ras-ERK) is known to regulate cell proliferation, differentiation, and survival 36-38. As many mammalian families of guanine nucleotide exchange factors impact the Ras activation cycle 39, this may be one mechanism by which RASGRF2 impacts STAD pathogenesis, and it is reasonable to speculate that targeting mechanisms of physiologic Ras activation may represent a novel approach in the treatment of KRAS-amplified cancers 40.

It has recently been proposed that tumor microenvironments, especially immune factors within such environments, play an important role in STAD progression 41, 42. Accumulating evidence indicates that tumor microenvironment immune subtypes can predict clinical responses to immunotherapeutic strategies across a variety of tumor types 41, 43. The present study demonstrated that high *RASGRF2* expression correlates significantly and positively with gene signature-estimated Tem, macrophage, pDC, and NK cell infiltration into the tumor. It is, therefore, reasonable to speculate that RASGRF2-associated immune genes or leukocyte infiltration patterns may provide similar prognostic value in predicting STAD response to immunotherapeutic interventions.

**Figure 7:**
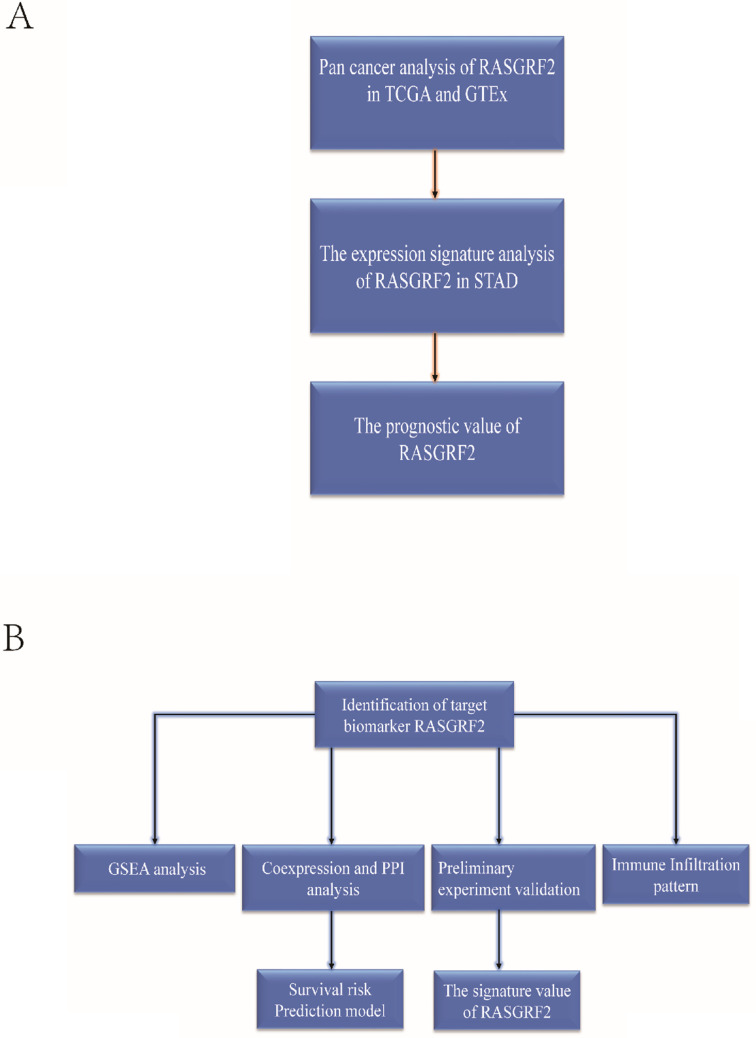
** The basic workflow of a comprehensive study is outlined. The study comprises two-part. (A)** The transcript expression signature, prognostic and diagnostic value of RASGRF2. **(B)** The functional value, tumor immune infiltration analysis, clinical use, and preliminary validation of RASGRF2 in STAD.

To sum up, this study is the first to investigate the transcript expression signature of RASGRF2, prognostic and diagnostic value, relationship with tumor immune infiltration, and associated functional pathways in STAD from the complementary bioinformatics. Furthermore, the RASGRF2 related survival prediction nomogram was developed and validated for predicting the survival probability of STAD patients. We also verified that RASGRF2 protein expression, correlation with clinicopathologic characteristics, and prognostic model performance in an independent STAD cohort. These findings verified the central role of RASGRF2 expression in STAD prognosis and tumor microenvironment, and shed light on a novel area for further exploration and confirmation (**Figure 7**).

## Conclusions

Complementary bioinformatics, *in vitro*, and clinical analyses suggest that the expression level of RASGRF2 may have diagnostic and prognostic value in the context of STAD. Candidate molecular mechanisms underlying this association include plausible interactions of RASGRF2 with the MAPK family, RAS, ERK, and immunoregulatory signaling pathways. Finally, RASGRF2-associated patterns of immune infiltration may help predict the response to immunotherapeutic interventions. Although our study was first to explore the comprehensive role of RASGRF2 in STAD, however, there is still a long way off to its clinical use. First, although the results of this study have been verified using clinical tissue samples and TCGA cohorts, the number of cases included is small. Second, a cell-level experiment can only carry out a preliminary verification of the protein expression level. Following this, we will continue to conduct further experiments to verify the biological function of immune function infiltration and the mechanism through which it affects the occurrence and development of gastric cancer.

## Supplementary Material

Supplementary tables.

## Figures and Tables

**Table 1:** Clinical characteristics of patients from The First Affiliated Hospital of China Medical University, and correlations between *RASGRF2* transcript level and clinicopathologic characteristics

Clinical characteristics of patients from The First Affiliated Hospital of China Medical University, and correlations between RASGRF2 transcript level and clinicopathologic characteristics				
Characteristics	Number of cases (%)	Low expression of RASGRF2	High expression of RASGRF2	*P* value
Tumor	72	11 (15.3%)	61 (84.7%)	
Adjacent non-tumor	72	10 (13.9%)	62 (86.1%)	
**strong positive expression (scores > 5)**				
Tumor	72	21 (29.2)	51 (70.8%)	
Adjacent non-tumor	72	38 (52.8)	34 (47.2)	
**Age (y)**				0.326
≥60	36 (50%)	7	29	
<60	36 (50%)	4	32	
**Gender**				0.968
Male	52 (72.2%)	8	44	
Female	20 (27.8%)	3	17	
**Clinical stage**				**0.009**
I	4 (5.6%)	2	2	
II	27 (37.5%)	7	20	
III	38 (52.8%)	1	37	
IV	3 (4.2%)	1	2	
**T stage**				**0.042**
T1	3 (4.2%)	1	2	
T2	10 (13.9%)	4	6	
T3	23 (31.9%)	4	19	
T4	36 (50%)	2	34	
**N stage**				0.349
N0	35 (48.6%)	8	27	
N1	10 (13.9%)	1	9	
N2	8 (11.1%)	1	7	
N3	19 (26.4%)	1	18	
**Metastasis**				0.166
No	70 (97.2%)	10	60	
Yes	2 (2.8%)	1	1	
**Histological type**				**0.032**
Papillary type	3 (4.2%)	0	3	
Tubular type	17 (23.6%)	6	11	
Poorly differentiated type	27 (37.5%)	5	22	
Signet Ring type	16 (22.2%)	0	16	
Mucinous type	9 (12.5%)	0	9	
**Venous invasion**				0.46254
No	70 (97.2%)	11	59	
Yes	2 (2.8%)	0	2	
**Lymphatic invasion**				0.339
No	43 (59.7%)	8	35	
Yes	29 (40.3%)	3	26	

**Table 2:** Univariate and multivariate Cox regression analyses incorporating clinicopathologic characteristics of patients from The Cancer Genome Atlas stomach adenocarcinoma dataset

Univariate and multivariate Cox regression analyses incorporating clinicopathologic characteristics of patients from The First Affiliated Hospital of China Medical University				
	Univariate analysis		Multivariate analysis	
	HR (95% CI)	*P* value	HR (95% CI)	*P* value
Age(y)	0.661 (0.322-1.357)	0.259	1.359 (1.141-1.911)	0.031
Gender	1.138 (0.5-2.593)	0.758	2.743 (0.965-7.802)	0.058
Clinical stage	0.516 (0.276-0.967)	0.039	1.092 (0.266-4.488)	0.903
T stage	0.618 (0.413-0.925)	0.019	0.870 (0.351-2.153)	0.763
N stage	1.643 (1.438-1.943)	0.024	1.549 (1.323-1.933)	0.026
Histological type	1.099 (0.774-1.559)	0.599	1.596 (0.971-2.623)	0.065
Lymphatic invasion	0.583 (0.254-1.335)	0.202	0.797 (0.313-2.028)	0.634
RASGRF2 (High vs. Low)	1.347 (1.145-1.828)	0.017	1.239 (1.069-1.823)	0.023

## Abbreviations

RASGRF2: Ras Protein Specific Guanine Nucleotide Releasing Factor 2
STAD: Stomach Adenocarcinoma
PPI: Protein-protein Interaction
DEG: Differentially expressed gene
TCGA: The Cancer Genome Atlas
GSEA: Gene Set Enrichment Analysis
MCODE: Molecular Complex Detection
IHC: Immunohistochemistry
